# An Overview of Additive Manufacturing of Polymers and Associated Composites

**DOI:** 10.3390/polym12112719

**Published:** 2020-11-17

**Authors:** Shukantu Dev Nath, Sabrina Nilufar

**Affiliations:** Department of Mechanical Engineering and Energy Processes, Southern Illinois University Carbondale, Carbondale, IL 62901, USA; shukantudev.nath@siu.edu

**Keywords:** additive manufacturing, polymers, reinforcements, auxetic metamaterials, triply periodic minimal surface structures

## Abstract

Additive manufacturing is rapidly evolving and opening new possibilities for many industries. This article gives an overview of the current status of additive manufacturing with polymers and polymer composites. Various types of reinforcements in polymers and architectured cellular material printing including the auxetic metamaterials and the triply periodic minimal surface structures are discussed. Finally, applications, current challenges, and future directions are highlighted here.

## 1. Introduction

Additive manufacturing (AM), also commonly known as 3D printing, is a process where parts are created layer-by-layer from 3D computer model data. 3D printing technologies involve a 3D object being sliced into computer-modeled layers followed by the deposition of each layer at a time. The term AM refers to the deposition of layer upon layer. AM was first introduced commercially in 1987 with stereolithography (SLA) by 3D Systems [1]. Since then, the AM has evolved very rapidly particularly in the last decade. Many techniques have been developed including fused deposition modeling (FDM; first introduced and commercialized by Stratasys), digital light processing (DLP; by Envision TEC), solid ground curing (SGC; by Cubital and Helisys), laminated object manufacturing (LOM; by Cubital and Helisys), selective laser sintering (SLS; by DTM Corp., a sister concern of 3D Systems) [1]. The printing technologies are classified in many ways in the literature. Some of them are classified according to the methods of deposition while others are classified according to the selection of materials [2,3,4]. Although the journey of AM began a few decades ago, standardization of different AM techniques has been done very recently. The AM processes can be classified into seven main categories [5], namely, (1) binder jetting, (2) directed energy deposition, (3) material extrusion (FDM, 3D plotting, micro extrusion), (4) material jetting (polyjet printing), (5) powder bed fusion (SLS, selective laser melting, electron beam melting), (6) sheet lamination (LOM), and (7) vat photopolymerization (SLA, DLP, two-photon polymerization). In this review, we will highlight some of the widely used polymer AM processes. Next, we will consider the materials available for polymer AM processes. The availability of a large variety of polymers in the form of resin, filament, and powder has become a popular choice for AM processes due to low cost of manufacturing particularly for a small class of low-range machines [6,7,8]. Furthermore, advantages like a smaller footprint and little to no maintenance for the high-end professional system of polymer printers have made them a popular choice in recent years. However, some AM systems may suffer from system instability thus requiring frequent system calibration. Thermoplastic polymers such as ABS (acrylonitrile butadiene styrene) and its derivatives [9,10,11], PLA (polylactic acid) [12], PVA (polyvinyl alcohol) [13,14], TPU (thermoplastic polyurethane) [15,16], nylon [17] are some of the commonly used materials for 3D printing. In addition, there is a growing interest to use materials of different stiffness ranging from soft, rubber-like materials to hard plastics in AM processes which could potentially be used in shoe soles, vehicle parts, and body armor [18,19].

Creating intricately shaped parts for research and commercial use can be expensive and difficult to fabricate. Before the AM revolution, thermoplastic parts were mostly made by injection molding. Nevertheless, injection molding is not profitable for small production lots. In contrast, AM has become a suitable and viable option for small production lots as it eliminates the cost of making molds [20]. Manufacturers are customizing their production using a combination of AM and injection molding [21]. Intricate parts can be fabricated with precision by AM processes nowadays. Thermo curable or photocurable acrylic and epoxy resins are among the most popular choices for printing intricate parts [22,23,24,25].

AM has found its way to myriads of applications [26]. Lightweight materials and structures manufactured by AM have potential use in marine and aerospace industries. The medical sector is utilizing the boon of AM by printing organs, tissues, blood vessel stents, etc. [27,28,29,30]. The dentistry found a way of printing accurate and custom-sized tooth, implants, and dental molds at a very reasonable cost [31]. Fabrication time also went down from weeks to several hours. Architects utilize AM processes to visualize and materialize their ideas. The cost of 3D printed buildings was reported to be cheaper than conventional construction methods. Recent NASA initiatives for MARS colonization have generated a great deal of enthusiasm for 3D printed habitats. Nevertheless, there are some serious limitations to AM. Many of these technical challenges still need to be addressed for meeting engineering demands such as customized geometry, building scalability, material heterogeneity, and structural reliability which are extensively discussed elsewhere [32,33,34,35]. This review paper aims to summarize the most recent works of AM focusing on polymers and polymer composites. Moreover, we will describe how the addition of reinforcements into the matrix materials can effectively improve the desired mechanical properties of the printed parts. Besides, we will highlight techniques for printing architectured cellular structures including the auxetics and the triply periodic minimal surface structures. Finally, we will discuss current applications, existing challenges, and future research directions of AM.

## 2. Selected AM Methods for Polymer and Reinforced Polymer Composites

### 2.1. Material Extrusion

#### FDM

The FDM printers are widely used and the most available printers for polymer printing. These printers utilize the filament extrusion method where the printing head melts the filament into a semi-liquid state, extrudes molten materials, and deposits single layers onto the build platform [36]. The commonly used filaments in FDM are thermoplastics such as ABS [9,10,11], PLA [12], PVA [13,14], TPU [15,16], ULTEM (polyetherimide) [37,38], nylon [17], ASA (acrylonitrile styrene acrylate) [39,40], and PPSF (polyphenylsulfone) [41]. The quality of printing can be varied by controlling several parameters including printing bed temperature, nozzle head temperature, nozzle size, layer thickness, printing orientation, printing speed, and raster angle [42]. Detailed studies of various processing parameters have been investigated elsewhere [43,44]. The study suggests proper bed temperature and controlled convective heat transfer conditions can lead to a higher bonding strength of the consecutive layers thus improve the mechanical property of the printed parts [43].

The main advantages of FDM are lower cost particularly for small footprint machines and multi-material object printing capabilities [45]. The material properties at different print locations can be optimized to exhibit different chemical and mechanical characteristics by using multiple extruders [46]. The limitations of the FDM process include the starting material to be in the filament form and the melting point of the filament cannot be very high except for some engineering polymers such as ULTEM and PEEK (polyetheretherketone) [38,47]. However, some micro extrusion systems eliminate the requirement to have the material in filament form altogether [48]. The possibility of the addition of different kinds of reinforcements may overcome some of these shortcomings. In addition, while printing with FDM, if the melt viscosity of the molten material is high, it may prevent extrusion and subsequently reduce printing speed. In contrast, if the melt viscosity is low, the print sections will fail due to its inability to hold unsupported sections thus creating structural instability [49]. Therefore, the range of melt viscosity of the material for FDM becomes limited. During the manufacturing of filament for FDM, the plastic is melted and extruded into a filament shape. While printing with the filament in FDM, the filament is again heated near the melting temperature. Therefore, it is critical for the filament to maintain its chemical stability and withstand physical strains regardless of temperature variations for high-quality printing [12,50].

### 2.2. Vat Photopolymerization

#### 2.2.1. SLA

Stereolithography utilizes the technique of curing photopolymer with an ultraviolet (UV) laser beam. The photopolymer is kept in a resin tank while the UV laser beam moves in the x–y plane and the printing base moves along the z-direction. There are two types of printing methods for SLA, namely, the top-down approach and the bottom-up approach. For the top-down printing method, a UV laser beam is pointed in a downward direction and runs along the design path on the surface of the resin. This action cures the resin and forms a layer of printing on the printing base kept at the desired depth from the resin surface. After the first layer is printed, the printing base is lowered down, and printing of the next layer starts as before. Entrapped uncured resin for some specific drawings could be a limitation of this method which can be avoided by carefully setting the printing orientation. For the bottom-up approach, a UV laser beam is directed towards an upward direction. The resin tank has an opening window at the bottom of the tank to allow the laser to pass through. The gap between the printing base and resin tank measures the layer height. UV light cures the resin under the printing base and after each layer is printed the printing base is raised according to the set layer height. Failure of base layer adhesion to the printing base limits the printing size in this process. A recent study discussed the processes and compared both “top-down” or “bottom-up” approaches [51]. According to the study, the bottom-up approach leads to more accurate and gives precise control over printing. However, detaching the printed layer from the printing bed subjects an unwanted cyclic loading on the printed part during printing. In contrast, the top-down printing approach does not apply any such loading during printing. Furthermore, very slender components become difficult to print and printing needs more support structures in the bottom-up approach compared to the top-down approach.

Acrylic and epoxy resins are typical materials used in SLA [3,52]. To make photopolymers, the photo-initiators are added to the resins [53,54]. Printed parts are cleaned and washed in acetone or isopropyl alcohol to dissolve the uncured resin residue found on the part surfaces. Cleaning time is carefully optimized to avoid adverse effects on the printed parts [55]. Controlling polymerization parameters including exposure time, UV intensity, speed of printing, layer thickness are very crucial to achieve proper printing with SLA [56,57,58,59,60]. Both top-down and bottom-up approaches provide very high resolution and accuracy [61]. However, this process is very slow compared to other methods. Slow printing speed and high resolution make SLA perfect for small precision parts.

#### 2.2.2. DLP

Like SLA, DLP also cures liquid resin using UV light. The only difference being the use of a digital UV projector screen to cure the photopolymers instead of a laser beam used in SLA. The screen illuminates the pixels according to each layer of the drawing. As a result, the whole layer is printed at once, unlike SLA, resulting in a much faster build speed.

The cured resin adheres to the printing bed one layer at a time. The screen solidifies the thin layer of resin entrapped between the printing base and resin tank base. After printing each layer, the base is uplifted to a distance allowing the liquid resins to fill up in-between space and then lowered down to a specific layer height. During the first few layers of printing, there is a suction between the printing base and the resin tank base. This may lead the layers to lose adhesion to the printing base creating printing failure. To avoid this, the uplifting speed of the printing base needs to be tuned carefully. The resolution of the prints directly depends on the pixel size of the digital screen, layer height [62], print layer exposer time [63], and bottom layer exposure time. The bottom layer exposure time is more relevant in the DLP printing approach where the bottom layer attaches to the print bed which is vital for successful printing. An additional post-curing process can be used to prevent the deformation of the thin printed parts [64]. In general, DLP has fewer moving parts and is much faster than SLA as it cures each layer all at once. A recent study demonstrated a system to print large models with a small DLP printer [65,66]. This system allowed to print models larger than the printing screen.

Both SLA and DLP are barely capable of printing objects below 10 µm. However, there is a growing demand for 3D micro/nano-AM techniques for many applications in the area of photonics, sensors, microelectromechanical systems, mechanobiology, and microfluidics [67,68,69]. Two-photon polymerization (2PP) is a micro AM process which is capable of printing parts as small as 100 nm. In this method, resins consisting of monomers or oligomers are modified with photoinitiators for generating radicals after excitation. Crosslinkers are also used to ensure the insolubility in the developing solvents [70]. Resins are polymerized not by absorbing one photon, but by simultaneously absorbing two photons at longer infrared (IR) wavelength regions. In some cases, cationic polymerization is also utilized. A cationic initiator transfers a charge to the monomer units to make them reactive. These activated monomer units lead to polymer chain growth [71].

In a very recent study, researchers have 3D printed the world’s smallest boat of 30 µm length. It is one third smaller than the thickness of a human hair and about six times larger than a bacterium. It has remarkable detailed features with an open cockpit featuring some complicated geometry. It will allow understanding of how micro-swimmers like bacteria and sperm move through liquids which will be useful for applications in therapeutic diagnostics and drug delivery [72]. 2PP accomplishes manufacturing that is otherwise not accessible and brings new scientific possibilities for the micro/nano AM research.

### 2.3. Powder Bed Fusion

#### SLS

SLS is a powder bed fusion process. A high-power laser beam is targeted over a dispersed thin layer of polymer powder inside of a build chamber. As a result, the powder is melted and fused, forming a layer of solid print. The binding mechanism of SLS technology can be discussed in three main sections [73]. (A) Complete melting where materials are fully melted using the laser beam. This is suitable for metallic and ceramic materials rather than polymers. Post-processing is seldom required for this kind of operation. (B) Liquid phase assisted sintering is commonly used for materials that are difficult to sinter. This is widely used for ceramic materials mixed with a small amount of polymer additives. (C) Solid-state sintering where the powder is not fully melted in the process but rather heated between melting temperature, T_m_ and T_m_/2. After the printing, post-processing is often needed [74,75,76].

SLS system has two powder tanks kept side by side. One is a feeder tank and the other is a printing tank. Both the chambers are kept at a temperature close to T_m_ of the powder. The laser beam moves along the x-y plane in a two-dimensional pattern and fuses the powder creating a printed layer. After printing of each layer, the powder bed is lowered according to layer thickness, while a new layer of fresh powder is spread over the previous layer from the feeder tank with the help of a slider or a drum. At the end of printing, the printed part is found in the printing tank and excess powder is removed. The excess powder material in the printing tank remains virgin and can be reused for the next printing. Polyamides (PA 11, PA 12) are the most used materials for SLS. The use of other polymers such as polyetherketoneketone (PEKK), TPU, PA 6 was commercialized with limited success [77]. The quality of the prints of SLS largely depends on the particle size, laser power, printing speed, and additional powder properties [78]. Unlike SLA, DLP, and FDM, SLS does not require structural supports for printing parts with overhanging features due to the presence of excess powder in the bed. However, post-processing is necessary for a better surface finish. The overall cost of the setup can be quite expensive because of high-power heating and high material waste [79].

### 2.4. Material Jetting

#### Polyjet Printing

Polyjet printing involves curing sprayed polymer with UV lights. A series of nozzles spray liquid or semi-liquid polymers to the printing bed. The sprayed polymer is continuously cured to a solid form with UV light. The series of nozzles are assembled in a gantry that moves in the x-y plane. The UV light source is also attached to the gantry. After printing each layer, the printing bed is lowered along the z-axis and makes room for the next layer of printing. Polyjet printing technology can print with any photopolymer if it is in liquid/semi-liquid form. The quality of the printing depends on the layer height, printing orientation, UV light intensity, and polymer spray rate [80,81,82,83]. The exposure time of the UV light is not variable as the UV is attached to the gantry that moves with the nozzles. Nevertheless, the exposure can be indirectly controlled by varying the rate of polymer spraying.

In polyjet printing, the printing process is very fast compared to other methods as multiple nozzles can spray polymers simultaneously. This also allows printing objects with multi-materials [84] at high-resolution [85,86]. Printed parts can be of different colors with a good surface finish [32] which makes this process ideal for rapid prototyping. Post-processing is not necessary for polyjet printing. The high cost of printing with a single material is a drawback of this process.

### 2.5. Sheet Lamination

#### LOM

LOM is a process of making 3D objects with the deposition of layers of laminated materials [87,88]. A sheet of plastic from a plastic roll (feed drum) is spread on the printing base. A laser moves in the x and y-axis and cuts the plastic according to the drawing. First, the laser cuts a rectangular area from the sheet and then cuts the drawing pattern on the layer. Next, any area outside the pattern is cross-hatched. This cross-hatching allows for easy removal of redundant materials at the end of the printing process. After each layer of cutting, a fresh sheet of plastic from the feed drum slides on the previous layer. The residue of the printed layer is collected in a residue drum. A heated hot leveler drum rolls on the new layer of plastic to adhere to the previous layer by heat activation. After each layer is printed the printing base is lowered down according to the sheet material thickness.

**Figure 1:**
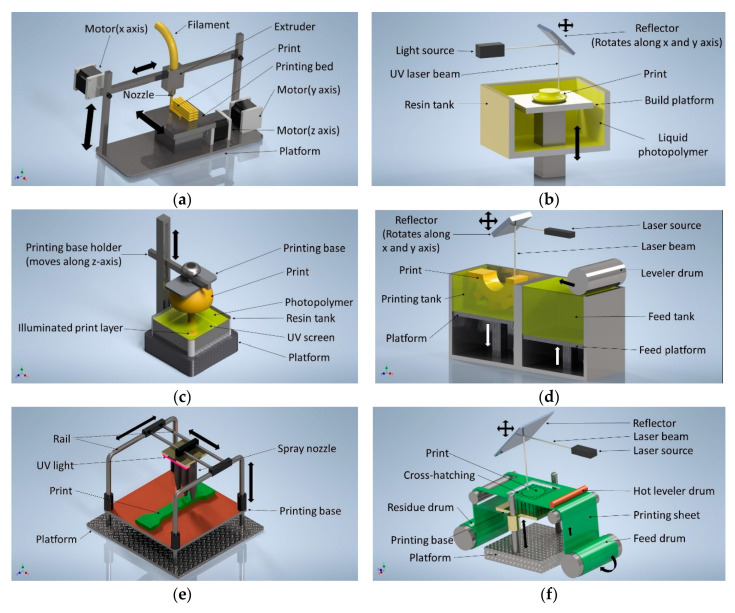
Selected techniques for polymer AM. (**a**) The Fused Deposition Modeling (FDM) printers work on the materials extrusion principle to print desired parts. (**b**) The stereolithography (SLA) technique uses a UV laser beam to cure liquid UV curable polymer for printing with high accuracy. (**c**) The digital light processing (DLP) printers print highly accurate parts using UV screens and are less time consuming than the SLA. (**d**) The selective laser sintering (SLS) fuses fine powder polymers with a laser beam. However, the printed parts produce rough or grainy surfaces. (**e**) In the polyjet printing process, fine drops of polymers are sprayed by multiple nozzles on the printing bed which are immediately cured by the UV light. It is capable of fast printing with multi-material deposition. (**f**) In the sheet lamination (LOM), sheets of polymers are precisely cut and added in layers to make the final product. When fast printing and large size printing capability are required, the LOM technique is preferred.

LOM is one of the widely used rapid prototyping methods [89]. It does not require any structural support for printing as the residual laminated sheet acts as a support for the print. With this method, only sheet materials can be printed. The layer thickness of the sheet plays a major role in terms of surface finish and the overall quality of the printed parts [90]. In general poor surface finish and low-resolution leads to post-processing for the printed parts [91]. The width of the sheet material rolls needs to be chosen carefully to minimize material waste. Printing complex parts with this method are not feasible because of the difficulty in removing redundant materials. Figure 1 displays different AM processes related to polymer AM.

Due to thermal expansion, the thermoplastic materials expand in volume during the printing process. After printing when the printed parts reach room temperature, the differential temperature leads to shrinkage in the final product. Such shrinkage may induce residual stresses in the printed parts which results in nonuniform and unpredictable mechanical properties. This issue can partially be addressed by choosing suitable materials that have minimal shrinkable behavior. Alternatively, the nominal dimensions could be larger than the actual dimensions to accommodate such shrinkage. To minimize shrinkage, Huang et al. [92] created a library based on the shape of the print that can anticipate shrinkage of the final product and adjust the drawing accordingly.

## 3. Reinforcements in 3D Printed Parts

Polymer 3D printed parts are weaker compared to conventionally fabricated parts. Stronger 3D printed parts are desired for load-bearing structures. Therefore, different types of reinforcements have been employed to serve this purpose. However, properly adding reinforcements remains a challenge in various printing techniques. Improving one property of the materials can negatively affect other properties. Past studies have utilized different reinforcement schemes for different processes. Reinforcements of polymer materials can be categorized into three sections, namely, (1) fiber reinforcements, (2) particle reinforcements, and (3) nanoparticle reinforcements.

### 3.1. Fiber Reinforcements for 3D Printing

Synthetic fibers such as carbon fiber (CF), glass fiber (GF), and Kevlar fiber provide excellent improvement of properties in the filaments and final parts. On the other hand, natural fibers are lightweight and cost-effective [93]. Natural fibers do not alter the biodegradability of the biodegradable polymers used in different composites. Selected fibers offer noteworthy improvement of different properties such as tensile modulus and strength, flexural properties, less distortion, shrinkage prevention, and acoustic and vibration attenuation properties. The most critical issue is the alinement of fibers in the printed parts for a given direction [94]. Among all 3D printing processes, fiber reinforcement has been extensively studied for the FDM process due to its ability to align fiber orientation. Many commercial vendors nowadays offer a wide range of fiber-reinforced filaments for FDM printing. In recent years, continuous fiber reinforcement allows introducing fibers directly into the polymer filament which eliminates the process of preparing fiber-reinforced filament for FDM printing. For example, Akhoundi et al. demonstrated that a high amount of fiber content into the PLA matrix showed improved modulus and strength of printed parts [95]. Many different continuous fiber reinforcement systems were also discussed in this study. In a separate study, a process of directly introducing fiber reinforcement into the thermoplastic filament while printing was discussed [96]. The process involves embedding a 7 µm diameter CF filament continuously into the melted polymer filament just before the deposition of the layers. The tensile modulus of the printed part showed more than 200% improvement while the bending modulus improved around 370%. The failure strain of the composite was less than that of pure PLA. Nowadays, many commercial FDM printers can print high-quality parts employing the continuous fiber reinforcement technique. Additively manufactured fiber reinforced parts have found their way in many applications such as replacement of metallic components in Formula SAE cars [97], parts in the Airbus A359XWB plane [98], reinforcement of cement mortars [99], etc.

Table 1 describes the outcome of fiber reinforcements in various polymer matrices.

In another study, CF, GF, and Kevlar fibers were introduced into nylon during printing [108]. The failure surfaces were also studied with the scanning electron microscopy (SEM) micrographs and relevant failure theories were discussed. The printed sample exhibited far superior tensile properties. Another group investigated the creep behavior of the same reinforcements in nylon [109]. Detailed fatigue analysis was conducted on the FDM printed part which shows both infill pattern and fiber volume fraction are critical to fatigue life of the fiber reinforced AM parts [110,111]. Different infill patterns (concentric, isotropic with 0°, 45°, and 90°) and different fiber volume fraction have a major impact on the fatigue life of the printed specimen. As the fiber volume fraction increases the fatigue performance increases. For the same fiber volume fraction, the 0° infill pattern showed the highest fatigue life.

Sometimes proper adhesion of the first layer to the printing bed becomes crucial for good quality printing when using polypropylene. The surface adhesion problem can be eliminated by adding short glass fiber [107]. Besides, short glass fiber also helps to reduce the shrinkage problem and the distortion of the final printed parts.

Natural fibers are also being utilized to improve the mechanical properties of printed parts. Depuydt and coworkers introduced flax and bamboo fiber into PLA [113]. They demonstrated that the aspect ratio of the fibers has a significant impact on the stiffness of the printed parts. They reported that a high aspect ratio of the fibers increased the stiffness of the parts by 215% compared to pure PLA while a low aspect ratio showed very minimal improvements. They extruded the fiber-reinforced PLA at different speeds. A low rotational speed allowed a high aspect ratio of the fibers in the PLA composite filament, though the surface of the filament turned out to be rough. The effect of the surface roughness needs to be studied furthermore in the future. Another example of natural fiber reinforcement involves hemp and harakeke fibers mixed into polypropylene matrix [119]. Both reinforced filament samples showed higher tensile strength and Young’s modulus compared pure polypropylene matrix.

### 3.2. Particle Reinforcements for 3D Printing

Particle reinforcements are becoming a popular choice for 3D printing primarily because of low cost and easy mixing with the polymer matrix. In general, particles are mixed with various polymer matrix for SLS or SLA processes and further extrusion of filaments for FDM processes. Depending on the size, filling factor (particle loading percentage), and material type, the characteristics of the resultant composites can be tailored accordingly. Some potential applications include printing soft actuators with stretchable piezo electrical composites, circuit boards with conductive particles, and multiple 3D transforming structures with ferromagnetic materials. Recent reports demonstrated the addition of metallic particles (steel and aluminum) of different filling factors into ABS filaments [120]. The smaller the particle size of metallic powder, the better the dispersion in the ABS matrix. In another study, the thermal conductivity of the printed composite was tuned with a higher percentage of stainless steel particles [121]. In addition to thermal properties, improvement in mechanical properties of the printed parts was achieved for up to 15 wt.% stainless steel particle reinforcement in ABS.

Technical ceramic particles like alumina (Al_2_O_3_) were added to various polymer matrices for 3D printing. Introducing Al_2_O_3_ into low-density polyethylene (LDPE) improved the mechanical properties of the printed parts [122]. Furthermore, dimensional accuracy and higher surface finish were achieved in the reinforced composites compared to the pure polyethylene matrix. In a separate study, the introduction of Al_2_O_3_ particles into the Nylon-6 matrix yielded better mechanical properties due to good dispersion into the matrix [123].

When magnetic particles are incorporated into the polymer matrix, the resultant composites can potentially act as a damper or barrier for shielding electromagnetic waves. Such composites have a great use for commercial and military applications. A recent study showed composite samples can be printed with 60–70% magnetite powder into epoxy resin and the samples showed improved compressive strength compared to neat epoxy resin [124].

The incorporation of lightweight hollow microspheres into the polymer matrix reduces the overall density of the composites [125]. Depending on the type of microspheres, the compressive properties of the composites can be tailored [126]. Dimensional stability can also be improved with the increase of microsphere content in the polymer matrix. 3D Printing with microsphere is a very recent endeavor. Singh and coworkers reported on adding fly ash cenosphere in a polymer-based filament for FDM printing [127,128]. The printed samples indicated an improvement in tensile properties. The same group also 3D printed composites from the cenosphere filled filaments and tested for compressive properties at various strain rates. The printed composites were lighter due to the presence of lightweight cenosphere and exhibited higher modulus at all strain rates [129].

The melt flow property of the material is an important factor for proper printing with the FDM process. When Al and ZrB_2_ microparticles were added to the ABS matrix, the melt flow index of the composite did not change significantly [130]. Due to the modification, a failure strain increase of ~80% for the ZrB_2_/ABS and 108% for the Al/ABS composites were observed.

In Table 2, the effects of different particle reinforcements in different polymer matrices have been summarized.

### 3.3. Nanoparticle Reinforcements for 3D Printing

The introduction of nanoparticles during AM processes is a major area of interest due to the possibility of various property enhancements including mechanical, chemical, electrical, and thermal properties of the printed parts [138,139,140,141,142,143,144,145].

Carbon nanotubes (CNT) can be used as an electrically conductive filler with photocurable resins [146,147]. Recently, a study showed the electrical behavior of CNT-filled 3D printable UV resin where the electrical conductivity of the printed parts was increased as a result of increased CNT content [148]. Several studies on the radar absorptivity of CNT-filled polymer composites have been conducted in the past [149,150,151,152,153,154,155,156,157]. In a recent study, radar absorbing composites were printed with SLA technology [158] where maximum absorption was found for 1.5% multi-walled CNT (MWCNT) filled composites. CNT-filled polyimide11 showed significant improvement in the toughness of the printed samples [159]. When a crack propagates inside a nanocomposite matrix, the CNT nanofillers work as bridges between the cracks, and additional energy is required to break and pull-out the CNT resulting in higher toughness.

Improved strength and modulus were observed for vapor-grown CF (VGCF) reinforced ABS composites printed by the FDM process [160]. However, the fracture mode of the composite changed from ductile to brittle with the addition of VGCF nanoparticles. Similar results were also reported for nanoclay/ABS and nanoclay/PLA composites made by the FDM process [9,161]. A very recent study with the FDM printing process reported different morphology and mesostructures associated with the addition of nanoparticles at different stages of printing [162]. The study indicates the mechanical properties of the printed part was improved when nanoclay was mixed with the ABS filament before printing. On-site mixing of the nanoclay decreased mechanical properties possibly due to the lesser extent of exfoliation and increased surface roughness but improved dielectric property. Lastly, the nanoclay coating after the printing resulted in lower mechanical properties but the surface roughness was drastically reduced.

Anisotropy of the printed material can be expected for all AM technologies [163,164,165,166]. Anisotropy limits the engineering applications of AM manufactured parts. Poor mechanical properties were observed in both y and z directions [167]. To address this issue, an innovative approach was introduced where each thermoplastic layer was coated with CNT [168]. With microwave heating, the interfaces between print layers were locally welded that allowed higher diffusion of adjacent polymer layers and increased fracture strength. An increase of 275% in fracture strength was reported over the conventional FDM printed parts.

In Table 3, the effects of different nanoparticle addition into different polymer matrices have been summarized.

The percentage of CF in a UV curable polymer should be low enough to have the proper curability of the composite and improved mechanical properties [169]. For SLA and DLP techniques, the refractive index of the printing material has a direct impact on the curability and curing time of the printing photopolymers. Added fillers in a composite may increase the mechanical, thermal, and electrical properties but the filler content and dimensional formation are crucial for the curing process. In a study, composites were filled with three silicon nanomaterials, namely, SiO_2_, ATP, and OMMT [174]. The addition of nanofillers with more than 10 wt.% made the composite unsuitable for printing with SLA. Interestingly, due to the zero-dimensional formation of SiO_2_ nanofillers, an increase of material properties was observed with little hindrance in the curing process. In contrast, one-dimensional ATP and two-dimensional OMMT hindered the curing process, thus affecting the material properties. Other studies also showed a minimal effect in cure rate and improvement in mechanical and thermal properties up to 10 wt.% (with minimum agglomeration) for SiO_2_ filled composites [9,172,175].

The dispersion of the nanoparticles in the base matrix is crucial for homogenous material properties. Without proper adhesion of the surfaces of the nanoparticles to the base matrix, agglomeration and sedimentation can occur. In this regard, surface treatment of the nanoparticles to promote proper adhesion to the matrix is necessary. Synthetic compounds such as silane coupling agents (SCA) are used to promote better adhesion between ceramic particles and the polymer matrix [176]. Several studies reported that SCA can be used as a coating on metallic nanoparticles to improve dispersion and surface adhesion to the polymer matrix as well. In 2007, Ukaji and coworkers studied the effect of SCA on TiO_2_ and found that the UV shielding ability was improved due to the treatment of particles [177]. In another study involving surface modification of TiO_2_ with SCAs showed better dispersion in a polyurethane matrix and improved mechanical properties [178]. Grafting an active functional group on the particle surface has been found to increase the bond strength of particle and matrix [179]. Grafting efficiency for two different SCAs on the TiO_2_ surfaces has been studied suggesting an improvement in particle dispersion stability [180]. The selection of SCA is a key factor for tailoring the surface characteristics of nanoparticles [181]. Improvements in mechanical and thermal properties, dispersion characteristics, effects of coating thickness for different metallic nanoparticles, and silane groups are reflected in many studies [138,182,183,184,185]. Recently, Sun and coworkers investigated the effect of SCA on zirconia nanoparticles [186]. They developed a relationship between cure depth and exposure dose of different silane group coated zirconia particles based on the polymer matrix. High shrinkage was observed in the prints but a layer thickness of 10 µm was achieved by the SLA process.

## 4. Selected Examples of Architectured Materials by AM Processes

AM processes allow the fabrication of materials with multidirectional tunable properties. In this section architectured cellular materials, namely, auxetic metamaterials and triply periodic minimal surface (TPMS) structures are discussed.

### 4.1. Auxetic Metamaterials

Auxetic metamaterials are created by a series of unit cells organized in such a way that the overall material expands laterally under tension and contracts laterally when subjected to compression. This unusual deformation feature of the auxetic structures gives rise to negative Poisson’s ratios. Auxetic structures have great potential for engineering applications as they are superior to conventional structures in many ways. Studies with auxetic materials reported high shear modulus [187,188], resistance to indentation [189,190], and improved fracture toughness [191,192] compared to conventional materials. 3D printing provides an ample opportunity to fabricate delicate shapes with intricate details. With 3D printing technologies, fabrication of such complex auxetic metamaterials became a reality and opened the possibility of more elaborate studies of auxetics to address future engineering demands. These auxetic metamaterials are not limited to polymers only, they can also be made with metal AM processes. Some of the practical applications of auxetic structure range from stents for angioplasty [193], shape memory alloy cellular antenna [194], footwear sole [195], and auxetic polyethylene in textile industries [196].

**Figure 2:**
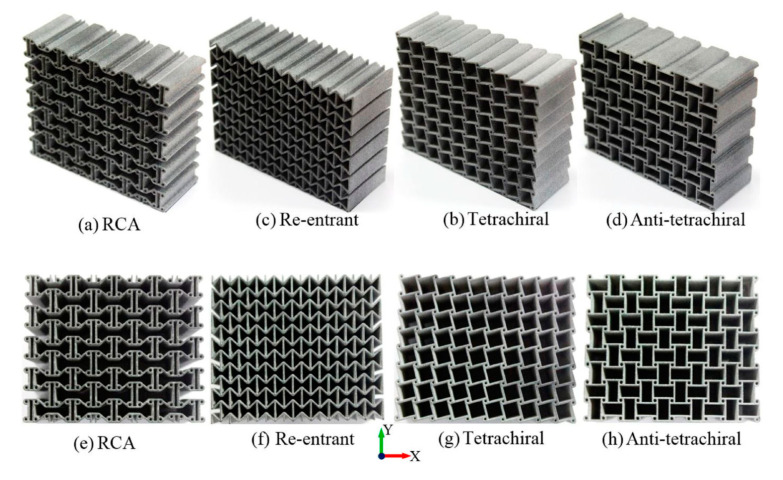
Auxetic cellular structures of different geometric shapes were printed with PA 12; (**a**,**e**) Re-entrant chiral auxetic (RCA); (**c**,**f**) Re-entrant; (**b**,**g**) Tetrachiral, (**d**,**h**) Anti-tetrachiral. Reprinted with permission from ref. [199]. Copyright 2020, Taylor and Francis.

A study of large deformation of 3D printed auxetic shapes using SLA showed the auxetic honeycomb to exhibit a very low negative Poisson’s ratio (as low as −4.0) [197]. Beyond that, the structure loses its auxeticity with further tensile deformation, and the Poisson’s ratio becomes positive. Auxetic shapes offer better energy absorption capacity compared to the conventional hexagonal or rectangular shaped structures. Different structures of auxetic metamaterials exhibit different characteristics. Using FDM, with a properly tuned relative density of auxetic shapes, up to a 33% increase in energy absorption has been reported [198]. A study with four types of auxetics using material jetting technique (re-entrant chiral auxetic, re-entrant honeycomb, tetrachiral honeycomb, anti-tetrachiral honeycomb; Figure 2) revealed that re-entrant chiral auxetic has the highest specific energy absorption capacity under quasi-static uniaxial load [199].

Though auxetic structures are better for energy absorption, these are less preferable when stiffness is under consideration. Auxetic structures are less stiff which restricts its usage to structural applications. A recent study with additively manufactured auxetic structure printed with CF reinforced polymer reported an increase of stiffness of their composite metamaterial with a decrease of the re-entrant angle of the structure [200]. With a relatively small re-entrant angle, they reported higher stiffness of the structure than that of steel. Future investigations on the alignment of the fibers can potentially improve the outcome.

Auxetic metamaterials can be tuned to specific properties. Shape memory polymers (SMP) can return to its original shape when the external force is withdrawn. Many studies reported the printing of auxetic shapes with SMPs to tune the properties of metamaterials. Yuan and coworkers thermomechanically tuned auxetics printed by polyjet process with 2-stage pattern switching [201]. Zhang and others applied heat and changed the FDM printed pattern using residual thermal stress [202]. Li and others applied the mechanical deformation of 3D printed (vat photopolymerization) PDMS membrane with a hot press [203]. Liu and coworkers demonstrated cylindrical auxetic structures fabricated by polyjet process with a zigzag pattern having isotropic Poisson’s ratio over a large range of strains [204,205]. With proper engineering, cylindrical auxetic shapes with SMP using DLP, can be used as medical stents [206].

### 4.2. 3D Printing of Triply Periodic Minimal Surface Structures

A triply periodic minimal surface (TPMS) is the surface of the minimal area between given boundary planes. These structures are quite fascinating, and the creation of such shapes has a rich history in mathematics. With the recent advancement in AM, TPMS generation has become an innovative way to meet engineering structural needs.

TPMS are non-self-intersecting cellular structures [207]. This family of architectured cellular structures gained special attention because of their unusual mechanical properties. TPMS has special and superior properties. Proper selection of TPMS structures can solve many engineering problems. Various TPMS geometric shapes have been shown to exhibit high specific stiffness [208,209]. Often TPMS geometries exhibit a large surface area which makes them ideal for their use as heat exchangers [210]. The TPMS structures printed by FDM can also provide heat insulation because of having porous structures [211]. Large deformation with compression or tension of different cellular structures helps absorbing more energy which makes them ideal for designing dampers. AM has opened a new door to study different architectured cellular geometries of TPMS. TPMS can also be beneficial for tissue engineering because of the tuning capability of pore morphology, cell interconnectivity, density, and toughness [212]. The studies of different complex shapes of TPMS structures were mostly investigated by computational simulations. Experimental studies were made possible only by the advancement in 3D printing technologies. One of the major problems of studying mechanical properties of different complex metamaterials is the discontinuity of the testing samples. Discontinuity induces stress concentration to certain areas which leads to offset or even inaccurate testing data. 3D printing processes allow TPMS structures to be continuous even for extremely complex shapes. Like auxetic metamaterials, TPMS is also not limited to polymer AM processes only. Metal AM processes also allow the generation of TPMS structures.

**Figure 3:**
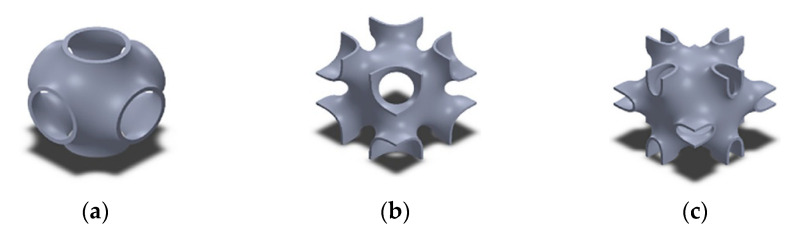

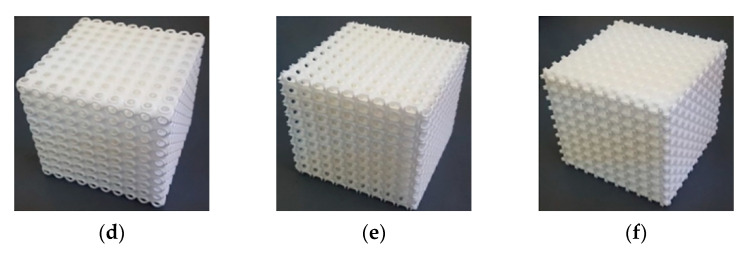
The first row shows computer-aided designs of gyroid structures while the second row shows corresponding printed cellular structures with a relative density of 10%. (**a**) Primitive-Cellular Materials (CM), (**b**) IWP (I-graph and Wrapped package-graph)-CM, (**c**) Neovius-CM. 3D printed specimens: (**d**) Primitive-CM (relative density is 23.5%), (**e**) IWP-CM (relative density is 25.6%), (**f**) Neovius-CM (relative density is 23.7%). Reprinted with permission from ref. [216]. Copyright 2017, Elsevier.

The gyroid structure is a special kind of TPMS known for high specific strength. NASA scientist Alan Schoen proposed a design for infinitely periodic minimal surfaces with no self-intersection back in the 1970s [213]. These structures can be found in nature. The butterfly wing scale is one of the perfect examples of gyroid structures [214,215]. Abueidda and others compared the mechanical properties of different kinds of additively manufacture TPMS structures printed using the SLS process (Figure 3) [216,217]. Among the structures, Neovius and IWP-cells showed the highest energy absorption rate. Primitive structures absorbed the least energy while the Gyroid structures were in the middle. With the increase of relative density, the structures were shown to absorb more energy [216]. For a given relative density, TPMS structures with a sheet-based TPMS network exhibits superior mechanical properties when compared to strut-based or solid TPMS structures printed by 2PP [218]. In addition, sheet-based gyroids printed by the SLA technique have been also reported to absorb more energy than the strut-based gyroids [219].

In addition, AM also makes it possible to fabricate functionally graded materials (FGMs) in which compositions and/or microstructures gradually change along with single or multiple spatial directions for enhanced performance.

## 5. Current Applications and Trends

### 5.1. Rapid Prototyping

Advancement in polymer AM has made a significant impact on rapid prototyping. Earlier, it was time consuming, expensive, and sometimes not even feasible to make prototypes of complex shapes. Engineers had to rely on computer modeling to design commercial products with limited capabilities of prototyping. AM opened the door for many kinds of prototyping with very little time and easy accessibility. Polymer AM technologies offer the opportunity to print very accurate models with marginal expenses compared to conventional prototyping processes. Moreover, any kind of design adjustments or adaptations can be incorporated with little effort.

### 5.2. Sensors and Electromechanical Systems

With the advancements of AM technologies and the availability of a wide variety of printing materials, AM is rapidly seeping into many sectors of engineering. One of the most benefited sectors is the electromechanical systems [220,221] and various other sensor design fields [222,223,224,225,226]. A recent study demonstrated the fabrication of a polypropylene-based thermoplastic composite for 2D and 3D circuits by FDM printing [227]. The printed circuits showed very low variability in electrical resistance in different environmental conditions. Another study reported a highly conductive liquid sensor by PLA-CNT nanocomposite using the FDM printing process [228]. Many engineering applications demand a flexible and complex circuit design. AM can tackle the printing of such flexible circuits with great ease [229].

The piezoresistive effect is a change in electrical resistivity with applied strain. FDM printed polymer matrix and CNT nanocomposite can be used to make strain sensors with tunable piezoresistive properties [223,224,225]. 3D printing of a multiaxial force sensor can eliminate the sequential steps of individual sensor fabrication followed by assembly steps [226]. With 3D printing, sensors can be printed directly without any sub-steps. A study demonstrated the ability to printing sensors and embedding it into a 3D printed part at the same time [222]. 3D printed temperature sensors showed excellent accuracy, various usability, and consistency [230,231].

### 5.3. Aerospace Industry

Metal AM has already made its way to the aerospace industry [232]. Different heavy-duty and functional engine parts are being manufactured by metal AM processes [233]. The aerospace industry started to embrace the convenience of design flexibility and reliability of metal parts made by AM. Likewise, polymers or polymer composites are being used in relatively low temperature areas such as fan duct, engine access panel, compressor vanes, fan bypass stator, and acoustic liner of engines [234].

PEKK and CF composite materials are high heat resistant, chemically inert, and mechanically strong. AM parts with the PEKK composite have potential use in the aerospace industry [235]. 3D printed ULTEM composites (family of PEI) are also good options for high-performance applications [236]. Polymer AM in unmanned aerial vehicles (UAV) is gaining popularity because of lightweight printed parts, ease of fabrication of aerodynamic shapes, and options for flexible design changes and/ or repair even in remote locations [237].

### 5.4. Automotive Industry

AM of polymer and polymer composites went from prototyping to production stage for automobile components such as car bumpers, windbreakers, interior/exterior trims using SLS AM processes. The supply of many spare parts for the automotive industry will be readily available as the AM technology matures [238].

### 5.5. Academic Institutions

One of the most benefited sectors of AM processes is education. Polymer 3D printing is cheap and easily available. It is being used to demonstrate ideas and model designs for K-12 outreach programs [239]. 3D printing has major implications in special education as well [240,241]. The importance and applications of 3D printing in human anatomy education are also vital [242,243]. 3D printing of molecular models can help the students visualize the structure more effectively [244,245].

### 5.6. Biomedical Applications

Polymers are widely used biomaterials for 3D printing. Both biodegradable and non-degradable polymers are available, but biodegradable polymers are widely used. Recently, many synthetic polymers have been developed with a tunable degradation rate. Hydrogels are a type of polymer that is used in a variety of applications including cell encapsulation, drug delivery systems, and scaffolds. Various 3D printing methods (FDM, SLA, DLP, etc.) have been adopted in studies to create scaffolds for specific tissues [246,247].

Drug development is benefited by AM processes. Drug dosage can also be tuned according to the prescriptions. Pharmaceutical companies are working on drugs which will be 3D printed with specific characteristics matching the individual patient dosage needs. FDM printing has found its way into drug research and development where thermoplastic polymers such as PVA have been utilized as drug carriers [248]. Researchers also demonstrated the capability of FDM in printing PVA filaments exhibiting an extended drug release of prednisolone for up to 24 h following oral administration [249]. However, a significant limitation for the use of FDM is the elevated temperature required for its operation, which may degrade a significant number of pharmaceutical excipients and active drugs [250]. Other water soluble polymers such as PVP (polyvinylpyrrolidone), and PAA (polyacrylic acid) are also used as excipients in drugs fabricated by AM [251].

Stents are made by the FDM technique using Silbione RTV 4439 A&B from Elkem Silicones, Oslo, Norway and polycaprolactone (PCL) is being used to prevent blocking or collapsing of blood vessels [252,253]. As the AM technology matures, we can fabricate and replace human organs using patients’ own cells that would minimize tissue rejection. Researchers have been able to fabricate prosthetics using AM processes [254,255,256]. AM processes enable to fabricate prosthetics to match the patient’s body structure. The quality and functions can be further adjusted.

### 5.7. AM Technologies in Dentistry

AM technologies have an obvious application in the field of dentistry. Many 3D printing companies specifically make 3D printers for prosthodontics applications. Polymers are mostly used to make the casting patterns for dentures. A complete denture or a part can be cast with metal from the printed patterns, which makes the process inexpensive, fast, and extremely precise. Ongoing research focuses on further material development to print dentures directly with 3D printing. Recently, the fabrication of artificial functional teeth with hard polymers and ceramics has become quite common [257].

Metal bracing was a popular option for straightening misaligned teeth. Discomfort, soft tissue injury, stuck food behind the braces, and overall appearances were among some of the issues with metal braces. Recently, 3D printed dental aligners utilize a series of clear and removable frames to gradually align the misplaced teeth [258]. These frames can be easily customizable for each patient according to their teeth size and shape. 3D printed dental models are being used for educational purposes as well.

Vat photopolymerization (SLA) technology is utilized in implant dentistry, as well as their applications in the field such as manufacturing surgical guides, custom trays, working implant casts, and provisional restorations [259].

### 5.8. AM in Vibration and Noise Control

Phononic Crystals are metamaterials with a periodic combination of scatterers in a matrix [260]. They exhibit the property of blocking frequencies of acoustic and elastic waves within a certain range, called a bandgap. For a given phononic crystal bandgap width, the frequency and isotropy depend on the topology, geometric structure, and properties of the materials of a unit cell [261]. AM has a huge advantage for the fabrication of phononic crystals and acoustic metamaterials. Resonators in the acoustic metamaterials can be achieved with different complex shapes, which are sometimes challenging to make with conventional methods. Conventionally fabricated phononic crystals and acoustic metamaterials lack tunable bandgap range which can be a big limitation [262]. AM leads the way to overcome this. 3D printing of the scatterers in the phononic crystals has become more common than before due to the advancement of AM technologies [263,264,265,266,267,268,269]. With the help of SLS, auxetic structures made of PA 12, was the first demonstration of a single-phase 3D periodic structure with wide tunable bandgap [270]. AM technologies are also utilized for improving the performance of acoustic metamaterials [271]. 3D printed samples of acoustic metamaterials were made by FDM, DLP, and SLA processes and subsequently compared the results with numerical modeling [271]. It was concluded that the divergence between numerical modeling and experimental results arises due to the difference in surface roughness of the printed samples.

### 5.9. Others

Manufacturing of eyewear frames is expensive as it goes through several steps such as modeling, cutting, heating, and reshaping, then polishing and assembling. AM can reduce some of the steps and make the design adjustments easier for the manufacturer. Among many, Fritz Frames Company is making some excellent AM printed glass frames. An interesting study indicated the security features offered by AM technologies by printing security labels with programable or customized geometry [272]. Under Armour and Adidas commercialized shoes with 3D printed soles. Carbon 3D introduced advanced helmet technology with 3D printed latticing support. These helmets can be customized according to the size and shape of the player’s head. A long ride on a bicycle can be very uncomfortable with an improper saddle. Extremely lightweight and comfortable saddles are made by using various AM technologies. AM processes are also utilized in making jewelry. Very recently, with the outbreak of the COVID-19 pandemic, AM technologies have been in use for 3D printing of face shields and masks to meet the market demand.

## 6. Challenges and Future Directions

AM processes allow design flexibility, customization, and rapid prototyping at an unprecedented level. The current application bubble is expanding at such a rapid rate that very soon it will affect all walks of life. From rapid prototyping to large scale manufacturing, certain challenges need to be addressed. First and foremost is the slow production speed of current AM systems. Therefore, there is a tremendous push for research and development of high-speed AM processes. Before the industry can adopt AM for large scale manufacturing, AM systems should be capable of producing parts in a matter of seconds to minutes rather than hours and days. The next big challenge is the availability of a narrow range of materials suitable for AM processes and the high cost of materials. Although the list of available materials is growing, it is not expanding fast enough and more importantly, there is no shared database of available printing materials and their properties. Along the line, materials used for AM processes are rather proprietary. This gives rise to another issue, i.e., heterogeneity in material properties, lot-to-lot variation, printed part variability, and quality control issues. Although AM processes are championed for less material waste compared to conventional processes, there is plenty of room for improvement in reducing materials waste. Furthermore, there is a vacuum of established industry-wide standards including guidance for green manufacturing. Moreover, design software availability, feeding the files for printing, and manual post-processing slows down the workflows. Finally, there is a size limitation. At one end of the spectrum, the manufacturing of large products becomes a bottleneck. On the other end of the spectrum, the printing resolution needs improvement. The bottom line, there is still a lot of room for future improvement to reap the full benefits of AM processes.

As for future work, we need to work towards reducing the gap between our current capabilities and expectation. Some of the major future directions are discussed here:

- The cost of polymer printed parts compared to conventional processes such as injection molding is higher. The ability to produce a large lot in a short period is the key to success. This will allow the transition from rapid prototyping to large-scale manufacturing in many industries. However, the use of AM processes does not need to compete with conventional polymer manufacturing processes rather the advantages of AM processes should be better utilized for finding solutions to unique problems.
- Low material cost, less waste, reuse of materials, low energy consumption, less manpower—all these factors can be combined to make the manufacturing greener.
- At present, the strength of printed parts can become a serious issue. This can be partially addressed by using reinforcements but still requires further research.
- The surface finish of printed parts is not satisfactory for some of the AM processes. The finish of prints with reinforcements is even worse. A better surface finish could eliminate the requirement for time-consuming post-treatment and save time and money.
- As most industries do not have a comprehensive R&D division, they tend to rely on vendors for any AM related processes. Therefore, the need for skilled workforce development in AM can be envisioned.
- Despite a vast application of 3D printing in different sectors, we are still limited to a very little variety of materials. The printing materials must be in filament, liquid, or fine powder form and cannot be reused once printed. This is a huge limitation for large-scale production. Synthesis of new materials with desired properties, new reinforcement capabilities, and reusable raw materials need to be explored further.

## 7. Conclusions

Polymers are base materials for majority of AM processes. Technological improvement and investigations in polymers as 3D printing materials is a fast-evolving field. Our goal is to put together a collection of very recent publications exploring various polymer AM processes related to reinforcements, different unusual cellular shapes, and highlight current accomplishments. AM has the potential to have a long-lasting impact in our day to day life but still have a long way to go to address the current challenges and drive towards future goals.

## Figures and Tables

**Table 1:** A summary of different fiber reinforcements into polymer matrices using FDM. ABS—acrylonitrile butadiene styrene; CF—carbon fiber; GF—glass fiber; PA—polyamides; PEEK—polyetheretherketone; PLA—polylactic acid; PP—polypropylene.

Matrix + Fibers	Property Evaluation	Remarks	Ref
ABS + CF	Composite with higher fiber length (150 µm) showed better tensile properties. 5 wt.% to 7.5 wt.% of fiber content in the composite had the highest tensile strength of around 43 MPa.	The mechanical properties of the final parts can be achieved at an optimum nozzle temperature of the extruder (~220 °C).	[100]
ABS + short GF	Improvement in tensile strength (~58.60 MPa) at the expense of flexibility and handleability.	The addition of plasticizer and compatibilizer recovered flexibility and handleability to some extent.	[101]
PLA + continuous CF	Around 200% and 370% increase in tensile and bending modulus, respectively.	Continuous CF reduces strain at failure. Annealing can increase the crystalline behavior of the composite.	[96,102]
PLA + CF	Improvement of tensile strength by 225% (28 MPa for pure PLA, 80 MPa for CF + PLA, and 91 MPa for modified CF + PLA) and flexural strength increased by 194% (53 MPa for pure PLA, 59 MPa for CF + PLA, and 156 MPa for modified CF + PLA).	The use of a coupling agent (methylene diacrylate) improves the adhesion between the fiber and the matrix.	[103]
PA 12 + CF	277.8% improvement of thermal conductivity of the composite was reported compared to pure PA 12.	The addition of CF increases the Melt Viscosity Index thus reducing the flowability leading to clogging in the extruder.	[104]
PEEK + CF	Improvement in tensile strength from 95 MPa to 101 MPa and modulus from 3.79 GPa to 7.37 GPa. Bending and compressive properties were also improved.	PEEK-GF composite showed comparable mechanical properties with human cortical bones. Surface roughness is ideal for biocompatibility, cell adhesion, and spreading.	[105]
PP + GF	0° filament orientation showed the highest modulus (~1.23 GPa) and strength (~35 MPa). However, the addition of GF resulted in a decreased melt flow rate.	Higher printing temperatures can rescue the reduced melt flow rate. However, the higher printing temperature may lead to distortion and shrinkage of the printed parts.	[106]
PP + short GF	Tensile modulus was improved (~400 MPa compared to pure PP ~75 MPa). Lower flexibility and elongation at break were observed.	GF reduced the shrinkage significantly. First layer adhesion and distortion of parts can be addressed by using GF.	[107]
Nylon + CF, GF, Kevlar fiber	Increased tensile strength (6.3 folds) and flexural strength (5 folds). However, the creep properties decreased.	Fibers were not embedded in the filament but printed as layers in between the Nylon layers in the parts. CF reinforced Nylon composite showed the highest improvements in the tensile strength.	[108,109]
Nylon + CF, GF, and Kevlar fiber	For any given infill pattern, progressively higher fiber volume fraction resulted in higher fatigue life. 0° orientation of isotropic infill pattern resulted in the highest fatigue life.	The 0° fiber orientation leads to high stiffness. The 45° fiber orientation makes the parts ductile. The loads are carried by the matrix only when the fiber orientation is set at 90°.	[110,111]
ABS + jute fiber	The tensile modulus was increased by 1% in horizontal printing direction and decreased by 25% in vertical printing direction with the addition of 5 wt.% jute fiber. Tensile strength decreased around 9% in both directions.	Jute fibers introduced more porosity in the filament. Decomposition of jute is observed during extrusion because of high temperature.	[112]
PLA + flax and bamboo fibers	High length to width ratio (l/d) of the fibers leads to increased stiffness (up to 215%).	The rotational speed of the filament extruder influences the *l*/d of the fibers in the filament. A higher *l*/d ratio of fibers in the filament makes the surface rough.	[113]
PLA + jute yarn	Improvement in tensile modulus and strength were observed by ~157% and ~134%, respectively.	Impregnation of fibers into filaments during extrusion resulted in continuous fiber orientation.	[114]
PLA + wood	The density of the printed composite decreased. However, the tensile strength did not change significantly.	The addition of a very high percentage of wood makes the surface rough.	[115,116]
PP + hemp and harakeke fibers	Tensile modulus and strength increased with the increase of hemp and harakeke fibers within the filaments.	With the increasing fiber content, the net shrinkage is reduced.	[117]
PP + bamboo fibers	Less than 500 µm sized fibers demonstrated the highest tensile strength (15 MPa for 50 wt.% fiber).	Bamboo reinforced PP composites are lightweight and water resistant.	[118]

**Table 2:** A summary of different particle reinforcements into polymer matrices. HDPE—high-density polypropylene; LDPE—low-density polypropylene; β-TCP—beta tricalcium phosphate.

Process	Matrix + Particle	Property Evaluation	Remarks	Ref
DLP	Acrylic based resin + microdiamond powder	The heat transfer rate was improved with 30 wt.% filler (30% of the time required compared to the pure matrix to reach the same temperature when heated), decreased thermal expansion coefficient, and decreased wettability.	More suitable for high temperature applications. High material costs may limit commercial use.	[131]
FDM	ABS + TiO_2_	Addition of 5 wt.% filler showed ~13.3% improvement in the tensile strength and ~11.6% improvement in the tensile modulus.	A smoother surface finish can be achieved by reducing voids which leads to better and consistent mechanical properties.	[112]
FDM	ABS + stainless steel particles	The tensile strength decreased slightly with the addition of stainless-steel particles. Specific heat increased to ~0.1 J/(g K) from ~0.05 J/(g K) (pure ABS) at constant pressure and at 200 °C.	Finer particles tend to disperse well. Defects and voids become dominant beyond a certain percentage of the particles which results in the decreased mechanical properties.	[121]
FDM	ABS + Al and ZnO_2_	Failure strain was increased by 80% for ABS/ZnO_2_ and 108% for ABS/Al.	The addition of metal and metallic powder did not change the melt flow properties significantly.	[130]
FDM	ABS + Cu and Fe	The tensile modulus increased by adding 10 wt.% of Cu (~930.2 MPa) and 30 wt.% of Fe (~978.5 MPa). However, the tensile strength was decreased by adding fillers. The thermal expansion coefficient of the composite with 50 wt.% Cu was decreased by 30% while thermal conductivity increased by 41%.	Strength reduces with the incorporation of fillers in the composite. The addition of Cu in the composite resulted in less distortion in the printed parts.	[132]
FDM	ABS + BaTiO_3_	Improvement of relative permeability was achieved by 260% at 35 vol.% while a 53% decrease in flexural strength was reported at 30 vol.%.	Inhomogeneous particle distribution may cause premature mechanical failure. Proper adhesion of the printed parts to the bed becomes challenging at higher vol.% of fillers (above 45%).	[133]
FDM	LDPE + Al_2_O_3_	The compressive strength was improved by 7%.	Better surface finish and dimensional accuracy can be achieved with Al_2_O_3_ addition into the polyethylene matrix.	[122]
FDM	HDPE + fly-ash cenosphere	Density was decreased and the tensile modulus was improved (2.6 times of HDPE filament), but fracture strain was decreased by about 40%.	Better quality of the printed parts can be achieved by optimizing the layer thickness, speed of the printer, print temperature, and cooling condition.	[127,128]
FDM	Nylon + Fe	Thermal conductivity increased by increasing vol.% and particle size of Fe.	The metal fillers form conductive particle chains in the matrix.	[134]
FDM	PA 12 + zirconia and β-TCP	The printed composites with 40 wt.% filler reported a tensile modulus of 995 MPa compared to that of pure PA 12 (~906 MPa). Tensile and flexural strengths decreased with increasing filler content.	Zirconia and β-TCP do not melt with the matrix during printing because of their high melting point. Agglomeration of the filler may cause clogging.	[135]
SLA	Epoxy resin + FeO	Printed parts with a layer thickness of less than 80 µm showed consistent mechanical properties. However, part thickness higher than 100 µm resulted in irregular properties.	SLA is a slow printing process. Therefore, micro particle-sized fillers must remain uniformly dispersed in the matrix for an extended period.	[136]
SLS	PA 11 + glass bead	The tensile modulus was improved with increasing vol.% of glass bead (~900 MPa for 10%, ~1250 MPa for 20%, and ~1750 MPa for 30%). The stiffness increased, but elongation at break was reduced.	The melting depth of the composite in SLS is crucial for successive layer adhesion. Printing settings should be optimized for different vol.% of glass beads.	[137]

**Table 3:** A summary of different nanoparticle reinforcements into polymer matrix for 3D printing. CNT—carbon nanotubes; SWCNT—single-walled carbon nanotubes; MWCNT—multi-walled carbon nanotubes; VGCF—vapor-grown carbon fiber; OMMT—organic montmorillonite; ATP—attapulgite; PEGDA—poly-ethylene glycol di-acrylate; Bis-GMA—bisphenol A-glycidyl methacrylate; TEGDMA—tetra-ethylene glycol di-methacrylate; GO—graphene oxide.

Process	Matrix + Nanoparticles	Property Evaluation	Remarks	Ref
DLP	Epoxy resin + MWCNT	The tensile strength increased by 5.7% (from 53 MPa to 56 MPa) and the flexural strength increased by 26% (from 42 MPa to 53 MPa).	Stable dispersion of MWCNT into the epoxy matrix is achieved by using only mechanical dispersion techniques. CNTs act as bridging component against the cracks.	[169]
DLP	PEGDA + MWCNT	The addition of 0.5 wt.% CNT resulted in higher electrical conductivity (~2 × 10^−5^ S/cm) compared to that of the pure matrix (~2 × 10^−9^ S/cm).	Dispersion issues are solved by the chemical dispersion technique.	[148]
DLP	Acrylic based resin + surface modified boehmite nanowires	The addition of 12 wt.% nanowire showed flexural strength of 125.7 MPa for Bis-GMA/TEGDMA resin (flexural strength for the neat resin is 113 MPa) and 11.3 MPa for PEGDA (flexural strength for the neat resin is 5.1 MPa).	Surface modification of boehmite nanowires is needed for proper dispersion. Addition of nanowires results in a smoother surface.	[139]
FDM	ABS + VGCF and SWCNT	The addition of 5 wt.% VGCF and SWCNT increased the tensile modulus by 44% and 93% respectively.	Poor surface interaction of VGCF with the matrix results in voids. High and uniform dispersion of the SWCNT without functionalization is possible which resulted in better surface finish and less voids.	[160,170]
FDM	ABS + Nanoclay	Addition of nanoclay before printing improved mechanical properties, on-site of printing improved dielectric properties, and coated with nanoclay after printing decreased surface roughness.	The addition of nano-filler in different stages of printing adheres differently with the matrix which changes the properties.	[162]
FDM	TPU + GO	Improvement of tensile modulus was achieved by 75.5% and compressive modulus by 167% with the addition of 0.5 wt.% of GO.	Mechanical properties can be tuned by adding different wt.% of GO filler in TPU. The TPU-GO composites can be used in cell culture studies because of the biocompatibility of TPU.	[167]
FDM	PLA + graphene	Tensile strength and modulus are reported 66.8 MPa and 3752 MPa respectively for PLA-graphene composites.	Graphene filler induces high anisotropy in the printed parts. Print direction must be optimized for higher mechanical properties.	[171]
FDM	PLA + MWCNT	The fracture strength increased by 275% compared to neat PLA printed parts.	Enhanced interlayer adhesion of the layers can be achieved with microwave heating.	[168]
FDM	PLA + Nanoclay	Storage modulus increased by 10% at 35 °C and 50% at 80 °C.	Higher thermal stability and degree of crystallinity can be achieved by adding nanoclay in the PLA matrix.	[161]
SLA	Acrylic ester resin + MWCNT	Addition of MWCNT increases the radar absorptivity.	The addition of filler is limited to 1.6 wt.% due to printability issues.	[158]
SLA	PEGDA + SiO_2_	The tensile strength increased with the addition of 1 wt.% silica. However, the tensile modulus showed an increasing trend with increasing amount (up to 5 wt.%) of silica nanoparticles.	Thermo-gravimetric analysis results showed inhomogeneous distribution due to the agglomeration of silica nanoparticle at higher loading.	[172]
SLA	Commercial resin-Clear V4 + SWCNT	The addition of 1 gm SWCNT in 1000 mL resin was reported in decreased impact resistance.	Local fracture in the SWCNT-polymer chain interfaces may lead to less impact resistance. Functionalizing the SWCNT may improve the results.	[141,173]
SLA	Acrylic based resin + SiO_2_, organic montmorillonite (OMMT), attapulgite (ATP)	0D SiO_2_ did not affect curing, but 1D ATP and 2D OMMT structures hindered the curing process.	Geometric formation of the fillers has a significant effect on the curability of the printed parts by vat photopolymerization. Viscosity of the resin is crucial for proper printability.	[174]
SLS	PA 11 + CNT	Impact strength increased ~54% with the addition of 0.2 wt.% CNT.	CNT causes to grow nano sized microcracks under loading instead of larger ones. Microcracks increase the total fracture surface area resulting in more energy absorption.	[159]
